# Transcriptome analysis of the spalax hypoxia survival response includes suppression of apoptosis and tight control of angiogenesis

**DOI:** 10.1186/1471-2164-13-615

**Published:** 2012-11-13

**Authors:** Assaf Malik, Abraham Korol, Mathias Weber, Thomas Hankeln, Aaron Avivi, Mark Band

**Affiliations:** 1 Institute of Evolution, University of Haifa, Haifa, Israel; 2 Institute of Molecular Genetics, Johannes Gutenberg University, Mainz, Germany; 3 W.M. Keck Center for Comparative and Functional Genomics, University of Illinois, Urbana, Illinois, USA

**Keywords:** Hypoxia, Spalax, Apoptosis, Angiogenesis, Cancer, Gene expression, Microarray

## Abstract

**Background:**

The development of complex responses to hypoxia has played a key role in the evolution of mammals, as inadequate response to this condition is frequently associated with cardiovascular diseases, developmental disorders, and cancers. Though numerous studies have used mice and rats in order to explore mechanisms that contribute to hypoxia tolerance, these studies are limited due to the high sensitivity of most rodents to severe hypoxia. The blind subterranean mole rat *Spalax* is a hypoxia tolerant rodent, which exhibits unique longevity and therefore has invaluable potential in hypoxia and cancer research.

**Results:**

Using microarrays, transcript abundance was measured in brain and muscle tissues from *Spalax* and rat individuals exposed to acute and chronic hypoxia for varying durations. We found that *Spalax* global gene expression response to hypoxia differs from that of rat and is characterized by the activation of functional groups of genes that have not been strongly associated with the response to hypoxia in hypoxia sensitive mammals. Using functional enrichment analysis of *Spalax* hypoxia induced genes we found highly significant overrepresentation of groups of genes involved in anti apoptosis, cancer, embryonic/sexual development, epidermal growth factor receptor binding, coordinated suppression and activation of distinct groups of transcription factors and membrane receptors, in addition to angiogenic related processes. We also detected hypoxia induced increases of different critical *Spalax* hub gene transcripts, including antiangiogenic genes associated with cancer tolerance in Down syndrome human individuals.

**Conclusions:**

This is the most comprehensive study of *Spalax* large scale gene expression response to hypoxia to date, and the first to use custom *Spalax* microarrays. Our work presents novel patterns that may underlie mechanisms with critical importance to the evolution of hypoxia tolerance, with special relevance to medical research.

## Background

The mole rat, *Spalax ehrenbergi* superspecies, is a wild subterranean rodent which lives in underground habitats, characterized by extreme hypoxic/hypercapnic conditions, and darkness [1]. Various chromosomal species of *Spalax* were identified, with diploid numbers ranging from 2n = 52 to 2n = 60. Phylogenetically, *Spalax* was suggested to belong to the Muroidea superfamily, and is closely related to Murine species (e.g., mice, rats). A *Spalax*-Murine common ancestor was suggested to live approximately 39 million years ago [2], during which *Spalax* acquired unique biological mechanisms to cope with environmental hypoxia, darkness, and other underground related stresses. Unlike various hibernating and diving mammals which experience short episodes of internal/environmental hypoxia, *Spalax* lives under chronic environmental hypoxia [3]. During the rainy season, the oxygen level in *Spalax* underground habitats was detected at 6% with CO_2_ levels around 7% [4]. In the laboratory, *Spalax* survives at 3% O_2_ for up to 14 hours, as compared to less than 4 hours for rats [4].

Although *Spalax* is phylogenetically close to rat and mouse, it differs in many aspects of metabolism, genetics, epigenetics, physiology and behavior. This species is, in most aspects, blind, with an impaired hearing at high frequency, like other subterranean species [5]. Compared with aboveground rodent species, *Spalax* has a higher density of blood vessels in muscle tissues, an increased lung diffusion capacity, and a higher erythrocyte count [6,7]. *Spalax* resting heart rate is about 40% of the expected rates for animals of similar size, reflecting increased aerobic capacity especially during tunnel system construction under hypoxic conditions [8]. Several hypoxia induced hub genes were found to exhibit unique expression patterns in *Spalax*, including Hypoxia inducible factor1-alpha (HIF1a), erythropoietin (Epo), and Epo receptors [9,10].

*Spalax* adults weigh 100–150 g, and can live at least 20 years in captivity. To the best of our knowledge, tumors have never been observed in *Spalax* wild or captive individuals, as compared to laboratory mice that tend to develop age related cancer. Similarly, another subterranean hypoxia resistant rodent, the naked mole rat, is considered to be cancer resistant and to exhibit unique longevity [11-13]. It was previously suggested that molecular pathways associated with hypoxia tolerance share common anti-apoptotic functions with those associated with tumor adaptivity [14-16]. Similarly, expression patterns of *Spalax* vascular endothelial growth factor (*Vegf*) are similar to those of tumors *Vegf*[6,17]. More generally, the association between hypoxia related and cancer related responses is based on wide evidence that tumor invasiveness requires cellular adaptation to hypoxic microenvironments [18]. Unlike the *Spalax* cellular response, hypoxic cancer cells acquire genomic instability [19].

We have used high throughput expression profiling to elucidate the response to hypoxic stress in *Spalax*. In an early study, a cross species microarray hybridization method was used for the detection of hypoxia induced expression patterns unique to *Spalax*[20]. More recently 454 technology was applied to sequence and assemble the *Spalax galili* transcriptome, using brain and muscle cDNA libraries created from pools of RNA extracted from individuals exposed to normoxia and hypoxia [21]. A total of about 50,000 *Spalax* contigs were assembled and mapped to more than 12,000 homologous mouse genes. 454 read count data was used for the detection of putative hypoxia induced *Spalax* genes.

In the present study, we utilized the newly sequenced *Spalax* genes [21] for the design of a custom *Spalax* microarray. Gene expression was measured in *Spalax* brain and muscle tissues from individuals exposed to different levels and time courses of hypoxia. More than 2,000 genes were found to be regulated during hypoxia in at least one tissue/treatment. We found a battery of biological processes/ontologies with significant over/under representation among hypoxia induced genes in *Spalax*. Here we report on the underlying biological processes and specific genes under regulation in hypoxic environments and potential hypoxia induced differences in expression patterns between *Spalax* and an above ground mammal, the rat.

## Methods

### Ethics statement

All animal handling protocols were approved by the Haifa University Committee for Ethics on Animal Subject Research, permit # 193/10 and approved by the Israel Ministry of Health. Permit # 193/10 covers all protocols and experimentation involving *Spalax*, rats or mice used in this experiment. This is a renewable permit which is current from July 2010– July 2014. The permit covers the number of animal subjects, housing conditions, veterinary regulations and inspections, hypoxia treatments and sacrifice methods for this experiment. No permits for capturing *Spalax* in unprotected areas are required. (Israel Nature Reserves Authority).

### Animals

*Spalax* were captured in the field and housed under ambient conditions in individual cages in the animal house of the Institute of Evolution. Animals were placed in a 70x70x50 cm chamber divided into separate cells where the chosen gas mixture was delivered at 3.5 l/min. Experiments were performed on adult animals of similar weight (100-150g) and included both genders. Three hypoxic conditions were chosen for *Spalax*: acute hypoxia of 3% O_2_ (4 animals), the lower limit of survival as determined in the lab, 6% O_2_ (3 animals), the level of oxygen measured in the field within *Spalax* tunnels after heavy rainfall [4] and mild long term hypoxia of 10% O_2_ for up to 44 h (5 animals), which is the estimated condition and time experienced by *Spalax* during tunnel reconstruction, as well as normoxia, 21% O_2_ (4 animals). Rats were exposed to either normoxic (21% O_2_) or hypoxic (6% O_2_) conditions (3 animals each condition).

### Tissues

Animals were sacrificed by injection with Ketaset CIII (Fort Dodge, USA) at 5 mg/kg of body weight. Tissues were removed and immediately frozen in liquid nitrogen. Brain tissues taken from individuals exposed to 3%, 6%, 10% and 21% O_2_, will be denoted br3, br6, br10 and br21, respectively. The corresponding hypoxic conditions for muscle tissues will be denoted mu3, mu6, mu10, mu21.

### RNA and cDNA preparation

Total RNA was extracted from whole brain or trapezius muscle tissues using TRI Reagent (Molecular Research Center, Inc., Cincinnati, OH) following the manufacturer’s instructions. RNA samples were treated with DNase I (Life Technologies, Grand Island, NY). All samples were tested for quality using an RNA nanochip on the Bioanalyzer (Agilent Technologies, Santa Clara, CA). 2 μg of total RNA were used for first strand cDNA using M-MuLV-H- reverse transcriptase (New England Biolabs, Ipswich, MA). The cDNA equivalent of 0.5 ng RNA was used in each real-time PCR reaction.

### Quantitative PCR mRNA quantification

mRNA expression levels were measured using real-time quantitative PCR (RLT-q-PCR). mRNA quantification was performed with three technical replicates (wells) for each sample using an ABI 7900 HT (Life Technologies) sequence detector in 384 well format. Gene expression was normalized to 18s rRNA. Primers were designed using Primer Express 2 software (Life Technologies) against *Spalax* transcripts. The primer sequences used for RLT-q-PCR are shown in Additional file 1: Table S1. Slopes of standard curves were: *DSCR1*: -3.2; *TSP1*: -3.22; *TNNC2*: -3.2; *TNNT3*: -3.19; *TNNI2*: -3.2; *18S*: -3.28. R^2^ was > 0.99 for all curves. All primer sets gave a single peak in the dissociation curves. Fold changes and significance levels were computed using REST software [22]. Significance was determined by permutation test using 10,000 iterations. Quantitative PCR was carried out on the same samples used for microarray analysis with the addition of 2 *Spalax* normoxic and 3 *Spalax* hypoxic 10% O_2_ brain samples available from previous experiments.

### Microarray probe design

Custom microarray probes were designed using contig sequences from the *Spalax galili* muscle and brain transcriptome assembly. In these datasets, contigs are annotated based on their predicted homology to mouse, rat, and human genes. Contigs/reverse complement contigs were aligned to homologous mouse Ensembl / Gene Bank transcripts, and probes were designed as close as possible to the 3’ end. In order to select the probes with the highest reliability, probes mapped to *Spalax* mouse conserved regions, and to large contigs were selected.

### Microarray labeling and hybridization

For each sample 200 ng of total RNA was labeled using the Agilent 2-color Low Input Quickamp Labeling kit (Agilent Technologys, Santa Clara, CA) according to the manufacturer's protocols. Labeled samples were hybridized to a custom designed *Spalax* 8 x 15K earray and scanned on an Axon 4000B microarray scanner (Molecular Devices, Sunnyvale, CA) at 5 um resolution. All microarray data files were submitted to Gene Ontology Omnibus (GEO) and are available for download with accession numbers: Platform, GPL15478; *Spalax* samples: GSM921638, GSM921946, GSM921979, GSM922315, GSM922635, GSM922638, GSM922639, GSM922640, GSM922799, GSM922977, GSM922978, GSM922979, GSM923030, GSM923039, GSM923051, GSM923052; Rat samples: GSM950599, GSM950600, GSM950716, GSM950717, GSM950718, GSM950719, GSM950770, GSM950865, GSM950940, GSM950947, GSM951034, GSM951035; Series GSE37619.

### Differential expression analysis

Spotfinding was carried out using GenePix 6.1 software (Molecular Devices, Sunnyvale, CA). Single channel differential expression analyses were conducted using Limma 3.10 [23], as follows: Background correction of signal intensities was carried out on GenePix data. Within array normalization, Loess, was performed on the background corrected signals, followed by quantile normalization between arrays. Limma separate channel analysis was conducted, and P-values adjusted for multiple testing were calculated by using the Benjamini and Hochberg method [24].

### Enrichment analysis

Three input lists were prepared: (1) a list of genes upregulated under the tested tissue/condition; (2) a list of downregulated genes; (3) a background list that includes all genes represented in the array. Before analysis, genes represented by probes with very low Cy5 and Cy3 emission intensities (<50) were removed from the three input lists. The IDs of mouse Ensembl genes mapped to *Spalax* contigs were used as an input. Gene Ontology (GO) enrichment analysis was conducted by comparing lists of hypoxia up/downregulated genes against the background list, using DAVID [25]. Similarly, the same procedure was repeated after removing entries linked to *Spalax* contigs mapped ambiguously to multiple genes. Association between enriched terms was calculated based on the size of overlap between sets of genes belonging to different terms, and visualized using Cytoscape [26].

### Heat Map generation

Heat map representation of differentially expressed genes was carried out with the R package Neatmap [27] using the average linkage algorithm.

## Results

### Estimating the scale of hypoxia induced differential expression in *Spalax*

**Figure 1:**
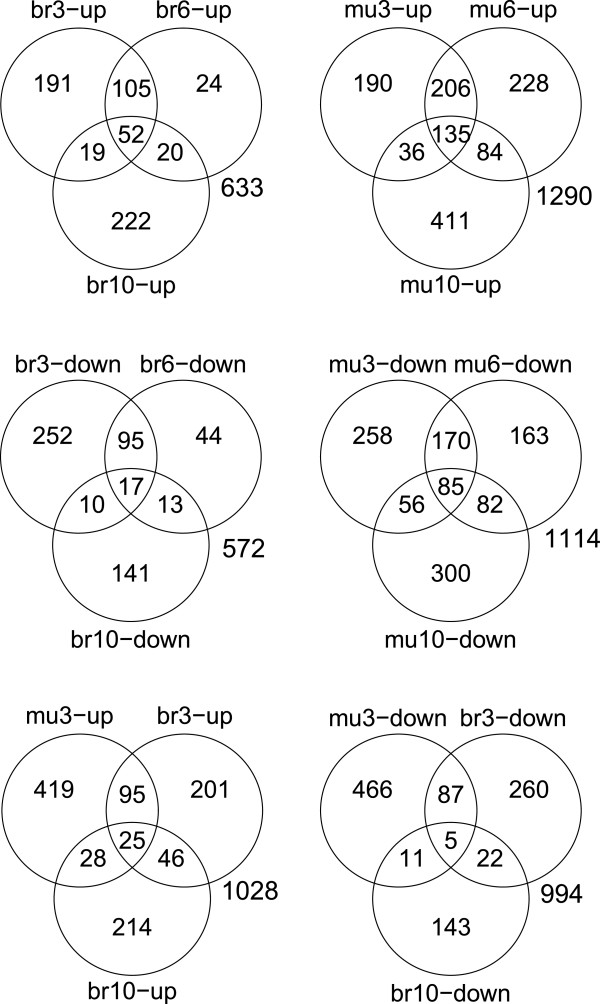
**Venn diagram of treatment specific hypoxia induced gene groups in *Spalax*.** Brain/muscle differential expression at 3% 6 hrs, 6% 6 hrs, 10% 44 hrs hypoxia, are denoted br3/mu3, br6/mu6, br10/mu10, respectively. Numbers inside closed regions represent counts of genes which are found to be differentially expressed as compared to normoxia. The numbers beside each diagram represent the union of the sets of differentially expressed genes.

**Figure 2:**
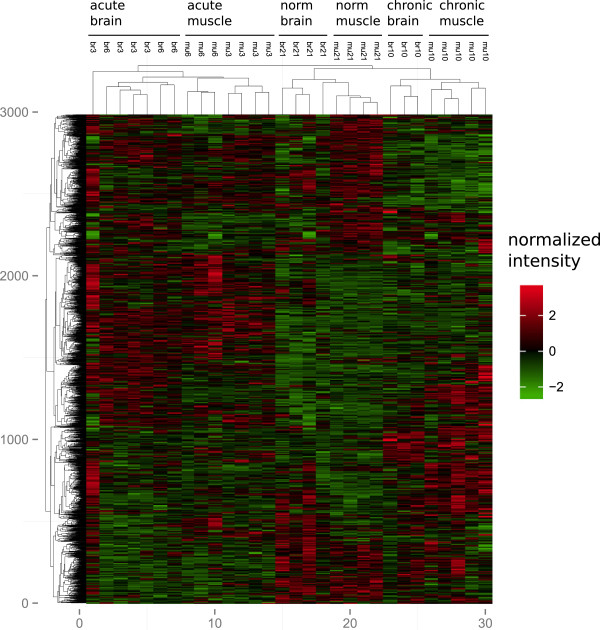
**Heat map representation of differentially expressed genes.** Based on microarray intensities, we performed hierarchical clustering of hypoxia induced genes and samples. The results indicate sharp differences between acute and chronic responses in *Spalax*, with clear tissue and treatment specific clades. In addition, it appears that acute hypoxia (3% and 6% O2) elicits similar responses both within and between tissues.

Based on multiple probe sequences designed from the *Spalax* brain/muscle transcriptome assembly we created a custom *Spalax* microarray using Agilent earray technology. *S. galili* muscle and brain transcript levels were measured in four experiments, under the following conditions: (1) 3% O_2_ for 6 hrs (2) 6% O_2_ for 6 hrs (3) 10% O_2_ for 44hrs (4) 21% O_2_ (normoxia). Experiment 1 (3% O_2_ 6 hrs) tests the response to extreme environmental hypoxia. This is the lowest oxygen concentration tested under laboratory conditions and is lethal to *Spalax* after >14 hours. Experiment 2 (6% O_2_ 6 hrs) aimed at detecting responses similar to those elicited under acute environmental hypoxia as measured in *Spalax* underground habitats after rainfall. Experiment 3 (10% O_2_ 44 hrs) tested responses under mild chronic environmental hypoxia. Under these conditions *Spalax* can conduct strenuous, energy consuming, tunneling work in its natural habitats. Differentially expressed genes were detected using Limma single channel analysis (Benjamini and Hochberg adjusted P-value <0.05) by comparing hypoxic to normoxic expression levels in brain and muscle (i.e., 3% vs. 21%, 6% vs. 21%, 10% vs. 21%). Limma differential expression statistics for all genes are shown in Additional file 2: Table S2. Responses to acute extreme hypoxia (i.e., mu3/6, br3/6) are profoundly different than responses to mild chronic hypoxia (i.e., mu10, br10) in terms of the number of genes affected in both types of responses (Figures 1, 2).

### Core responses to hypoxia in *Spalax* brain and muscle

**Figure 3:**
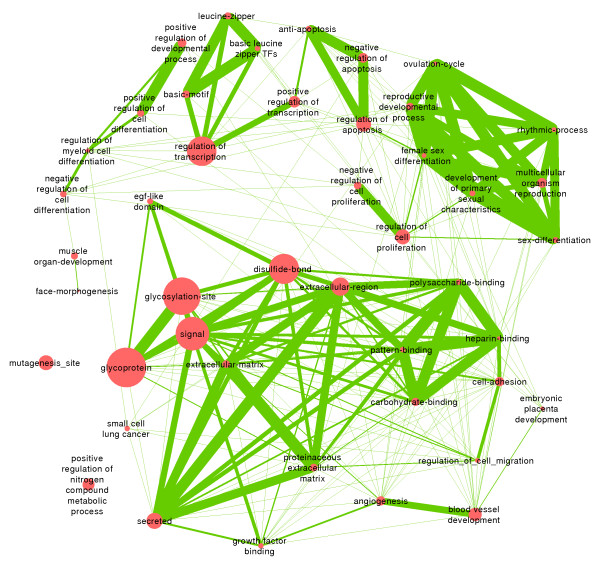
**Clusters of significantly enriched functional groups among genes upregulated under hypoxia in at least 3 experiments.** Nodes (circles) represent enriched gene ontology terms. Node size corresponds to the number of genes mapped to the term. Different pairs of terms (i.e., nodes) are connected by edges, where increased edge thickness corresponds to larger pairwise similarity, reflecting the proportion of shared genes.

As Figures 1 and 2 demonstrate, multiple genes exhibit similar response to hypoxia in both *Spalax* muscle and brain, and in both 3% and 6% hypoxia. These results may indicate that similar processes are activated under different conditions of hypoxia in *Spalax*. We further tested which types of processes are specifically associated with groups of genes expressed in more than one tissue/treatment. Accordingly, we selected groups of *Spalax* genes up/down regulated under hypoxia in ≥1, ≥2, and ≥3, out of six experiments (i.e., mu3/6/10, br3/6/10), as test groups. The test groups were compared to a background group composed of all genes represented on the array. Multiple processes were detected with highly significant enrichment among hypoxia responsive genes Additional file 3: Table S3. We assessed the biological similarity between enriched terms and it appears that many terms represent related processes. Accordingly, Figure 3 shows network representation of the biological association between terms enriched at adj. p-value < 0.05, among genes upregulated in at least 3 experiments. For example, as the figure indicates, terms representing different extracellular and transmembrane domains are associated with each other, as well as those representing cellular migration functions, growth factor binding, non-canonical EGF receptor (EGFR) ligands, and angiogenesis.

**Figure 4:**
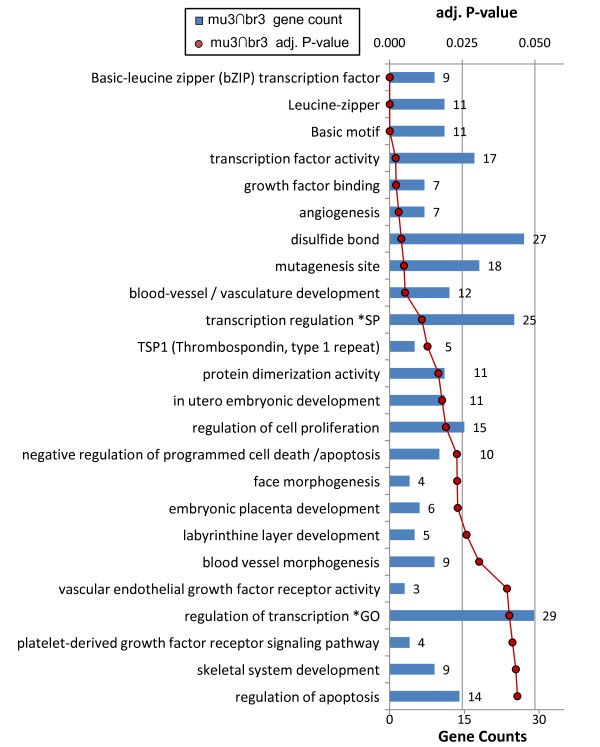
**Functional groups significantly enriched among genes upregulated in both *Spalax *muscle and brain after 3% 6hrs hypoxia (mu3∩br3).** Red line chart represents Benjamini adjusted P-values for specific ontology categories. Blue bars denote gene counts. A total of 75 genes are associated with at least one significant group. Enrichment of the same term may be detected using different databases (e.g. GO, Swiss-prot keywords, InterPro, Smart, KEGG).

We further tested responses activated under extreme hypoxia in both muscle and brain. As a test group we selected all *Spalax* genes that were differentially expressed in both brain and muscle (adj. p-value <0.05) under 3% hypoxia for 6 hrs (br3∩mu3 gene set). Of the 120/92 genes that were up/downregulated, 75/28 genes were associated with at least one significantly enriched Gene Ontology term (adj. p-value <0.05) (Figure 4, Additional file 4: Table S4). Collectively, these results indicate very strong enrichment of multiple groups of terms, including some exceptional terms such as ‘negative regulation of apoptosis/cell death’ and ‘basic-leucine zipper (bZIP) transcription factors’ (TFs). Table 1 summarizes the lists of genes associated with core hypoxia ontologies.

**Table 1:** ***Spalax *hypoxia induced genes belonging to selected enriched terms**

**Blood vessel development**		**Enzyme linked receptor**		S1pr1	2	Icam1	2	Fntb	3	Gna13	2
				Plaur	2	Serpine1	2	Btg1	4	Pecam1	6
				Nrip1	2	Tlr4	2	Loc100046643	4	Ppara	3
Acvrl1	5	Acvrl1	5	Fzd4	2	C3ar1	2	Zbtb16	5	Serping1	2
Col18a1	3	Ltbp1	2	Arntl	2	Serpina5	4	**Positive regulation of cell differentiation**		Tollip	2
Pgf	2	Tiam1	4	Kdr	5	Oasl1	2			F5	2
Hmox1	2	Axl	2	Thbd	4	Plaur	2			F3	2
Epha2	2	Epha2	2	**Egf like**		Cfi	2	Tiam1	4	Tnfrsf1b	2
Ctnnb1	2	Arid5b	3	Ltbp1	2	B2m	4	Igfbp3	6	Pf4	2
Cav1	3	Ctgf	3	Lamb1-1	3	Cxcl12	4	Id2	3	Cxcl1	4
Ctgf	3	Vegfa	6	Epha2	2	Thbd	4	Tnfrsf12a	4	Tgfbr2	2
Gna13	2	Nrp1	2	Hyal2	3	Bnip3	4	Cd74	3	Myd88	2
Trp63	3	Eng	3	Lama2	3	**Negative regulation of apoptosis**		Inpp5d	4	Fntb	3
Hes1	2	Skil	2	Vcan	2			Ets1	5	Nfkbiz	4
Adamts1	5	Egf	2	Plau	3			Btg1	4	Tlr4	2
Tnfrsf12a	4	Pdgfra	5	Thbs2	4	Angptl4	6	H2-aa	4	Loc640441	3
Vegfa	6	Tgfbr3	3	Itgb1	2	Bcl2l1	3	Foxo3	3	Plaur	2
Nrp1	2	Flt1	6	Lamc2	2	Btg2	3	Junb	3	Gja1	3
Eng	3	Eif4ebp1	2	Pamr1	3	Bnip3l	2	Socs3	5	Cfi	2
Dll4	2	Adam9	2	Dll4	2	Myc	5	**Brlz**		Sphk1	3
Egf	2	Tgfbr2	2	Cd93	3	Trp63	3	Mafk	3	Thbd	4
Cyr61	3	Myd88	2	Egf	2	Cdkn1a	4	Bach1	3	**Sex dev.**	
Nr4a3	2	Csrnp1	4	Pear1	4	Vegfa	6	Atf3	4	Acvrl1	5
Nos3	3	Tiparp	3	Tnc	2	Pim1	3	Fosl2	3	Lamb1-1	3
Pdgfra	5	Smad6	2	Mfge8	3	Cd74	3	Maff	5	Pafah1b2	3
Tgfbr3	3	Plekha1	2	Adam9	2	Snca	2	Junb	3	Ctnnb1	2
Flt1	6	Ltbp4	2	Eltd1	4	Csda	2	Cebpb	5	Cav1	3
Mgp	2	Pik3r1	3	Loc640441	3	Bag3	2	Cebpd	6	Bcl2l1	3
Mfge8	3	Foxo1	5	Ltbp4	2	Tsc22d3	4	Mafb	3	Sirt1	3
Adm	3	Kdr	5	Clec14a	3	Agt	2	**C2h2 like**		Spata9	3
Col4a1	5	**Receptors**		Ptgs1	2	Gclc	2	Rnf166	3	Dab2	6
Col4a2	5	Acvrl1	5	Thbd	4	Cited2	3	Zkscan17	3	Trp63	3
Cdh5	3	Axl	2	Heg1	2	Pik3r1	3	2810021j22rik	2	Adamts1	5
Agt	2	Cd36	2	**Growth factor binding**		Foxo1	5	Hinfp	5	Vegfa	6
Robo4	3	Gab2	2			Cebpb	5	Zfp661	4	Sf1	3
Tgfbr2	2	Pgrmc1	3			Sphk1	3	Zfp39	3	Itgb1	2
Tiparp	3	Epha2	2	Ltbp1	2	Bnip3	4	Zfp750	2	Xrn2	3
Twist1	2	Hyal2	3	Htra1	3	Prnp	3	Zfp691	3	Ccna1	2
Btg1	4	Mertk	2	Igfbp4	2	**Negative regulation of cell differentiation**		Gm6766	4	Cyr61	3
Adamts2	2	Scara5	2	Ctgf	3			Zfp512	2	Tnc	2
Loc100046643	4	Ednrb	2	Igfbp3	6			**Krab**		Nos3	3
Cited2	3	Osmr	3	Eng	3	Ctnnb1	2	Zkscan17	3	Pdgfra	5
Loc640441	3	Ppara	3	Cyr61	3	Cav1	3	2810021j22rik	2	Tgfbr3	3
Zc3h12a	2	Il15ra	3	Pdgfra	5	Sirt1	3	Zfp661	4	Csda	2
Foxo1	5	Tnfrsf12a	4	Tgfbr3	3	Nfkbia	4	Zfp39	3	Tead4	3
Shb	4	Itgb1	2	Col4a1	5	Trp63	3	Pogk	4	Adm	3
S1pr1	2	Nrp1	2	Ltbp4	2	Hes1	2	**Btb / btb poz**		Sfrp1	2
Gja1	3	Fcgr2b	3	Kdr	5	Rcan1	4	Kctd2	2	Agt	2
Junb	3	Mrc1	2	**Immune Response**		Cd74	3	Zbtb4	3	Egln1	3
Socs3	5	Slc20a1	2	Vwf	2	Itgb1	2	Rhobtb1	2	Cadm1	2
Rhob	4	Cd93	3	Gadd45g	4	Nrp1	2	Btbd11	2	Klf9	4
Cxcl12	4	Trpc4	2	Cxcl14	3	Inpp5d	4	Rhobtb2	2	Adamts2	2
Sphk1	3	Ptgfrn	4	Plau	3	Skil	2	Loc100046682	3	Siah1a	4
Kdr	5	Nr4a3	2	Bnip3l	2	Wwtr1	6	Ipp	2	Slc30a1	5
**Ecm receptor interaction**		Tnfrsf1b	2	Serping1	2	Tgfbr3	3	Klhl8	2	Cited2	3
		Errfi1	5	Vegfa	6	Pf4	2	Kbtbd8	2	Serpina5	4
		Pdgfra	5	Cd74	3	Rnf6	2	Klhdc5	3	Maff	5
Vwf	2	Tgfbr3	3	Tollip	2	Twist1	2	Zbtb38	2	Nrip1	2
Lamb1-1	3	Flt1	6	Inpp5d	4	Pik3r1	3	Kctd21	4	Foxo3	3
Cd36	2	Robo4	3	F5	2	Zfp36	2	Gm6766	4	Aff4	2
Sdc4	2	Tgfbr2	2	Fcgr2b	3	Zbtb16	5	Gm14457	3	Gja1	3
Lama2	3	Lpar1	2	F3	2	Cav1	3	Zbtb12	2	Junb	3
Thbs2	4	Tlr4	2	Tnfrsf1b	2	Igfbp3	6	Spop	2	Socs3	5
Itgb1	2	Eltd1	4	Tinagl1	4	Cdkn1a	4	Loc638050	2	Kdm3a	3
Col5a2	2	Pnrc1	3	Pf4	2	Sf1	3	**Wounding**		Cebpb	5
Lamc2	2	C3ar1	2	Cxcl1	4	Inpp5d	4	Klf6	4	Cxcl12	4
Tnc	2	Chrnb1	2	Cadm1	2	Fcgr2b	3	Vwf	2	Ppp1r1b	2
Col4a1	5	Cmklr1	3	Myd88	2	Nos3	3	Entpd2	2	Kdr	5
Col4a2	5	Gm5898	2	Sbno2	2	Tgfbr3	3	Igfbp4	2	Zbtb16	5
Loc640441	3	Cxcr7	5	H2-aa	4	Cdh5	3	Map2k3	4	Thbd	4

The number of conditions in which significant over/underexpression was detected in the microarray is shown to the right of the gene symbol, for 215 different genes. The list is based on Additional file 3: Tables S3 and Additional file 4: Table S4 for genes with unambiguous annotations. Group titles in bold, represent specific functional terms or groups of synonymous terms. *Sex dev.* Denotes reproductive development. *Enzyme linked receptor* denotes enzyme linked receptor signaling pathway. *Brlz* denotes basic leucine zipper TF.

### Processes suppressed during hypoxia in *Spalax*

**Figure 5:**
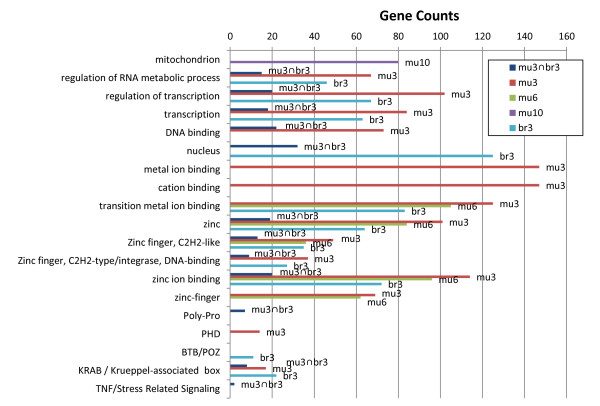
**Functional groups significantly enriched among genes downregulated in *Spalax *under hypoxia.** Bars represent gene counts for the different gene ontology terms.

In order to detect processes that may be suppressed under hypoxia, groups of genes downregulated in hypoxia were compared to all genes tested in the array. Groups of TFs, including zinc fingers and C2H2-like zinc fingers, are significantly overrepresented among genes downregulated in mu3, mu6, br3, and br3∩mu3 (Figure 5, Additional file 3: Tables S3, Additional file 4: Table S4, Additional file 5: Table S5). In addition, the Krüppel associated box (KRAB) subgroup of C2H2 Zinc finger TFs is overrepresented among genes downregulated in mu3, br3, and br3∩mu3. These results indicate that the repression of zinc finger TF groups is accompanied by the upregulation of several other TF groups under acute extreme hypoxia. Though these TF groups include multiple paralogs, where many probes mapped ambiguously to the mouse genome, similar results are also found after excluding such cases (Additional file 3: Tables S3, Additional file 4: Table S4, Additional file 6: Table S6). Collectively, hundreds of TFs are found to be enriched among genes suppressed under hypoxia. In addition, genes encoding mitochondrial proteins, and/or include transit peptide domains, are significantly overrepresented among downregulated genes in mu10 (see Figure 5). In most experiments, test groups composed of genes identified to be down regulated with adjusted P-value < 0.25, show highly significant enrichment of mitochondrial genes (e.g. enrichment adjusted P-value < 10^-24^ in mu10), as well as genes encoding electron transport chain proteins, oxidation/reduction proteins, and proteins located in the inner mitochondrial membrane (results not shown). Though the use of this liberal P-level for the test group selection might lead to non-negligible false positive detection, when sets of randomly selected genes of the same size were taken as test groups, no significant terms were found. Accordingly, the forgoing enrichment of downregulated mitochondrial genes should be noted, however additional bioinformatic tests will be needed for validations.

### Response to acute hypoxia in *Spalax*

**Figure 6:**
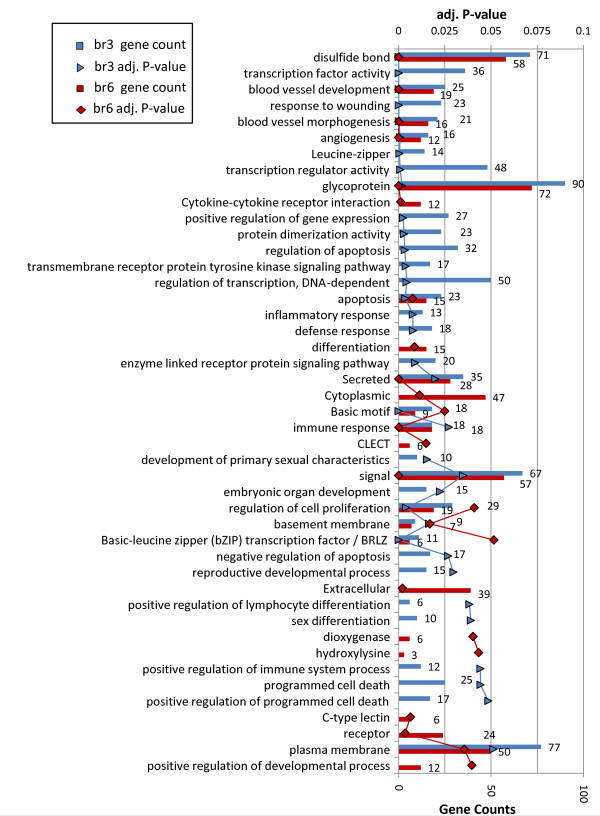
**Functional groups significantly enriched among genes upregulated in *Spalax *brain under acute hypoxia (br3, br6).** Series names: brain upregulation in hypoxia for 3% 6 hrs, and 6% 6 hrs, are denoted br3 (blue), br6 (red), respectively. Line chart (triangles, diamonds) represents Benjamini adjusted P-value of term enrichment. Bars denote gene counts.

**Figure 7:**
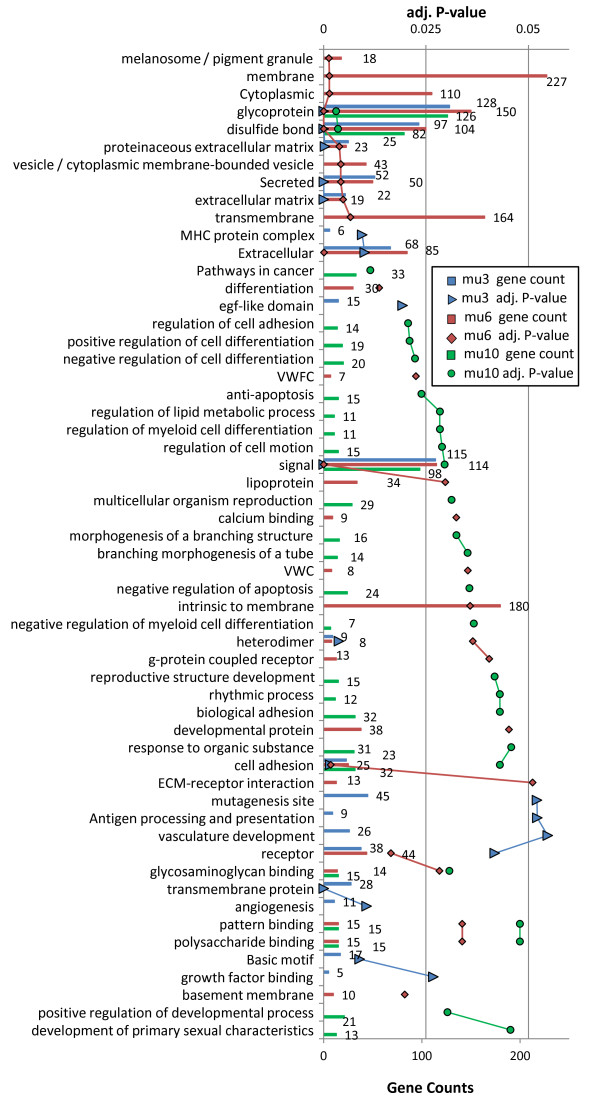
**Functional groups significantly enriched among genes upregulated in *Spalax *muscle under acute and chronic hypoxia (mu3, mu6, mu10).** Series names: muscle upregulation at 3% 6 hrs, 6% 6 hrs, 10% 44 hrs hypoxia, are denoted mu3 (blue), mu6 (red), mu10 (green), respectively. Line chart (triangles, diamonds, and circles) represents Benjamini adjusted P-value of term enrichment. Bars denote gene counts.

A total of 258, 360, 216, and 129 genes were mapped to significantly enriched functional terms among 567, 653, 367, and 201 genes significantly upregulated in mu3, mu6, br3 and br6 respectively (Figures 6 and 7, Tables S5 and S6). In general, most of these enriched terms are found to reoccur, or to correspond to analogous terms, in more than one experiment. These include terms that are angiogenesis related (in br3, br6, br10, mu3), disulfide bonds, glycoproteins, and signal peptides (in br3, br6, br10, mu3, mu6), bZIP TFs and regulation of transcription (in br3, br6), extracellular matrix binding and adhesion related (in br10, mu6, mu10), immune response related (in br6, br10, mu3), regulation of apoptosis related (in br3, mu10), and transmembrane proteins (in br3, br10, mu3, mu6), among many others. In addition, different groups of terms are found to be experiment specific, such as the mu6 specific term ‘cytoplasmic vesicles’ and its descendent term ‘melanosome’ (Figure 7). While melanosomes are not formed in muscle, many genes expressed in melanosomes have powerful antioxidant functions [28]. These results possibly reflect an overlap between gene sets involved in protective pathways induced by UV stress with those induced by production of radical oxygen species in hypoxic muscle.

### Responses to chronic hypoxia

Terms found to be significantly enriched in mu10 include: ‘pathways in cancer’, ‘negative regulation of cell death’, ‘negative/positive regulation of cell differentiation’, ‘regulation of cell adhesion’, ‘reproductive process’, and others (Figure 7). Collectively, these terms correspond to processes that contribute to cell survival under an internal state of hypoxia (e.g., in tumor microenvironments). Yet, it should be noted that with the exception of cancer related terms, most of the other terms in mu10 did not remain significant after removing genes with ambiguous annotations, though many had adj. p-value <0.1 (Additional file 6: Table S6).

**Figure 8:**
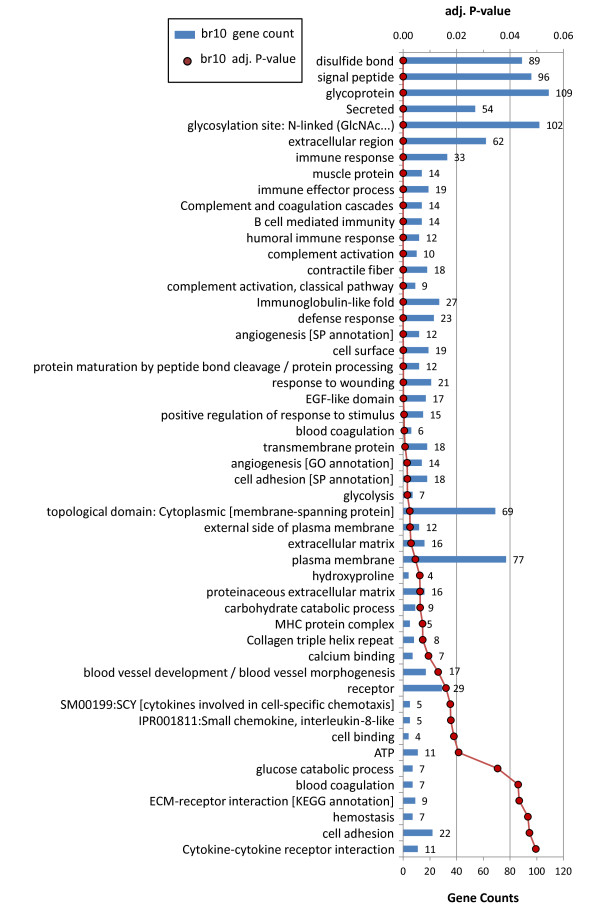
**Functional groups significantly enriched among genes upregulated in *Spalax *brain after 10% 44hrs hypoxia (br10).** Red line chart (scale on the top) shows Benjamini adjusted P-value for specific ontology terms. Blue bar (bottom scale) denotes gene counts. The listed terms were first clustered, so that similar categories are typically adjacent.

Very strong enrichment patterns were detected in br10 (Figure 8). In addition to core terms enriched in most other conditions, several terms appear exclusively in br10, including different immune response related terms, glycolysis, and ’contractile tissue’ related terms. As many of these genes are not typically regulated in brain tissues we verified this response using quantitative PCR with additional samples.

### Comparing *Spalax* and rat expression under 6% O_2_

Using a rat specific microarray, gene expression was measured in brain and muscle tissues from rat individuals exposed to 6% O_2_ for 6 hrs. The same basic microarray procedures were used in both species (see Methods). We did not expose rats to lower oxygen levels as such exposure becomes lethal within 2–4 hours. Similar to *Spalax*, angiogenesis and adhesion related terms are enriched among hypoxia induced genes, though the total number of responsive genes was found to be lower than *Spalax* (132/35 up/down regulated in rat br6, and 219/139 up/down regulated in rat mu6). We identified hundreds of genes that respond to hypoxia in only one of the two species (adj. p-values <0.05 vs. >0.5), among these, 66 genes with strong microarray signals (>200, for the non-responsive probe), and high fold change differences (>2 or <0.5) (Table 2 and Additional file 7: Table S7). These 66 genes include different tumor or angiogenesis suppressors such as *Rcan1* (upregulated by >10 fold in *Spalax* mu6), and apoptotic genes such as *G0s2* (downregulated by 25 fold in *Spalax* mu6). *G0s2* is a pro-apoptotic gene, and its observed downregulation may contribute to cell survival under hypoxia in *Spalax*. It is possible that some of the above differences reflect variation in probe sensitivity between rat and *Spalax* microarrays, but we still expect that strong differences have true biological repercussions in most cases.

**Table 2:** **Differences in hypoxia induced expression between *Spalax *and rat genes**

**Fold/Fold**		**Tissue**	**Symbol**	**Rat Gene**	***p.adj***	***Fold***	***i***	**p.adj**	**Fold**	**i**
spalax/rat	4.1	mu6	Cd44	ENSRNOG00000006094	1.3E-03	3.8	626	9.3E-01	0.9	227
spalax/rat	2.9	mu6	Htra1	ENSRNOG00000020533	1.9E-04	3.5	865	5.3E-01	1.2	433
spalax/rat	3.0	mu6	Myc	ENSRNOG00000004500	9.6E-06	5.1	319	6.4E-01	1.7	303
spalax/rat	2.8	mu6	Dirc2	ENSRNOG00000002240	9.9E-04	2.3	627	7.3E-01	0.8	939
spalax/rat	9.2	mu6	Rcan1	ENSRNOG00000001979	9.3E-05	11.5	4651	7.4E-01	1.3	1362
spalax/rat	3.6	mu6	Cdkn1a	ENSRNOG00000000521	9.8E-04	4.1	534	5.9E-01	1.2	6085
spalax/rat	2.5	mu6	Synj2	ENSRNOG00000017114	5.6E-05	3.2	376	6.8E-01	1.3	1070
spalax/rat	22.5	mu6	Tnfrsf12a	ENSRNOG00000003546	3.1E-05	13.8	3805	6.7E-01	0.6	479
spalax/rat	5.5	mu6	Uimc1	ENSRNOG00000016891	2.4E-03	6.3	27190	8.9E-01	1.1	494
spalax/rat	2.0	mu6	Mall	ENSRNOG00000015599	1.4E-02	2.3	524	7.4E-01	1.2	295
spalax/rat	2.8	mu6	Fuca1	ENSRNOG00000009325	5.3E-03	2.4	961	9.9E-01	0.9	1251
spalax/rat	5.7	mu6	Csrp3	ENSRNOG00000014327	2.9E-04	4.2	146	5.2E-01	0.7	6760
spalax/rat	4.5	mu6	Tyrobp	ENSRNOG00000020845	2.0E-03	3.6	1382	6.5E-01	0.8	395
spalax/rat	2.9	mu6	Bag3	ENSRNOG00000020298	9.8E-04	3.6	21589	9.1E-01	1.2	4629
spalax/rat	5.9	mu6	Pank1	ENSRNOG00000018944	4.0E-04	4.4	451	5.2E-01	0.7	329
spalax/rat	2.2	mu6	Btg1	ENSRNOG00000004284	2.1E-04	2.1	1075	9.2E-01	0.9	1426
spalax/rat	2.7	mu6	Rbm24	ENSRNOG00000016925	4.1E-04	2.5	4026	1.0E+00	0.9	201
spalax/rat	3.9	mu6	Mical2	ENSRNOG00000016244	1.1E-02	3.6	263	9.9E-01	0.9	200
spalax/rat	3.5	mu6	Cpeb2	ENSRNOG00000005043	2.2E-04	2.6	315	6.9E-01	0.7	959
spalax/rat	3.7	mu6	Cited2	ENSRNOG00000012193	2.9E-05	7.5	2023	8.5E-01	2.0	334
spalax/rat	4.9	mu6	Hspb8	ENSRNOG00000022392	3.2E-04	3.3	31123	5.8E-01	0.7	771
spalax/rat	6.7	mu6	Abra	ENSRNOG00000007999	1.3E-03	5.1	25220	6.6E-01	0.8	5289
spalax/rat	3.2	mu6	Cxcr7	ENSRNOG00000019622	2.6E-03	4.4	1227	5.0E-01	1.4	321
spalax/rat	3.6	mu6	Lrrc30	ENSRNOG00000030389	9.1E-06	4.0	3162	9.9E-01	1.1	406
spalax/rat	2.3	mu6	Prnp	ENSRNOG00000021259	4.0E-04	2.4	904	9.7E-01	1.0	1019
spalax/rat	7.7	mu6	Neu2	ENSRNOG00000016962	8.4E-04	4.0	145	7.0E-01	0.5	816
spalax/rat	2.8	br6	Hyal2	ENSRNOG00000031420	5.5E-04	2.9	1212	1.0E+00	1.0	1895
spalax/rat	2.4	br6	Igfbp3	ENSRNOG00000008645	4.2E-04	2.5	254	9.5E-01	1.0	696
spalax/rat	13.6	br6	Glt8d1	ENSRNOG00000018179	4.2E-04	11.7	497	7.6E-01	0.9	485
spalax/rat	2.7	br6	Kcnc2	ENSRNOG00000004077	2.5E-02	3.1	129	7.8E-01	1.1	1518
spalax/rat	3.9	br6	Junb	ENSRNOG00000042838	6.2E-04	2.5	2276	6.7E-01	0.6	4618
spalax/rat	0.02	mu6	G0s2	ENSRNOG00000006019	6.5E-05	0.04	3960	8.7E-01	1.6	11657
spalax/rat	0.2	mu6	Rgs2	ENSRNOG00000003687	7.0E-03	0.3	627	6.7E-01	1.7	618
spalax/rat	0.3	mu6	Akr1e1	ENSRNOG00000017165	4.8E-03	0.2	552	8.7E-01	0.8	765
spalax/rat	0.3	br6	Cnksr3	ENSRNOG00000018052	5.0E-02	0.4	1561	8.6E-01	1.3	209
spalax/rat	0.5	br6	Dek	ENSRNOG00000016152	2.1E-02	0.4	2266	7.7E-01	0.9	2232
spalax/rat	0.4	br6	Dhrs4	ENSRNOG00000018239	1.6E-02	0.5	6739	8.0E-01	1.1	253
spalax/rat	0.5	br6	Golga4	ENSRNOG00000029910	7.7E-03	0.5	4883	9.8E-01	1.0	4198
rat/spalax	2.0	mu6	D930049a15rik	ENSRNOG00000010488	7.5E-01	1.1	336	3.7E-03	2.3	221
rat/spalax	18.1	mu6	Ccng1	ENSRNOG00000003256	9.8E-01	1.0	13045	3.0E-02	18.0	6981
rat/spalax	3.5	mu6	Lman2	ENSRNOG00000016161	9.3E-01	1.0	262	5.5E-03	3.5	5729
rat/spalax	2.8	mu6	Tfrc	ENSRNOG00000001766	7.6E-01	0.8	2688	2.5E-02	2.1	107
rat/spalax	4.2	mu6	Slc25a25	ENSRNOG00000014338	9.9E-01	1.2	2496	2.4E-02	5.0	6771
rat/spalax	3.4	mu6	Pde4b	ENSRNOG00000005905	6.9E-01	1.1	479	1.2E-02	3.8	248
rat/spalax	2.2	mu6	Iqcg	ENSRNOG00000026420	7.5E-01	1.2	640	3.7E-02	2.7	159
rat/spalax	4.1	mu6	Loc100045963	ENSRNOG00000009028	7.9E-01	1.0	359	1.0E-02	4.0	3102
rat/spalax	4.6	mu6	Myf6	ENSRNOG00000004878	7.7E-01	0.7	1077	3.0E-02	3.3	3508
rat/spalax	4.5	mu6	Txnip	ENSRNOG00000021201	7.9E-01	1.1	12024	6.8E-03	4.9	6996
rat/spalax	3.3	mu6	D0h4s114	ENSRNOG00000020467	8.3E-01	0.9	1720	4.8E-02	3.0	699
rat/spalax	5.8	mu6	Junb	ENSRNOG00000042838	5.5E-01	1.5	1300	8.5E-04	8.7	3795
rat/spalax	2.5	br6	Pla1a	ENSRNOG00000027252	5.8E-01	1.1	448	2.5E-03	2.7	372
rat/spalax	2.2	br6	Cnp	ENSRNOG00000017496	7.7E-01	1.0	13280	1.5E-02	2.3	1677
rat/spalax	2.8	br6	Fn1	ENSRNOG00000014288	9.8E-01	0.9	937	1.9E-03	2.6	1577
rat/spalax	2.6	br6	Mfsd2a	ENSRNOG00000014008	9.1E-01	0.9	738	5.5E-04	2.4	703
rat/spalax	3.5	br6	Alas1	ENSRNOG00000000167	8.9E-01	1.0	2449	2.1E-03	3.6	1689
rat/spalax	0.4	mu6	Znf512b	ENSRNOG00000015558	6.8E-01	0.9	259	5.2E-03	0.4	1730
rat/spalax	0.4	mu6	Ier3	ENSRNOG00000000827	7.5E-01	1.1	1509	8.2E-03	0.4	281
rat/spalax	0.5	mu6	Ezh1	ENSRNOG00000020336	9.7E-01	1.1	1965	4.0E-02	0.5	2366
rat/spalax	0.4	mu6	Dynll1	ENSRNOG00000011222	5.1E-01	0.9	22039	1.9E-02	0.4	2236
rat/spalax	0.4	mu6	Rpap1	ENSRNOG00000005483	5.1E-01	1.1	575	2.6E-03	0.4	249
rat/spalax	0.4	mu6	Ypel3	ENSRNOG00000019721	8.4E-01	1.0	2745	6.0E-03	0.4	769
rat/spalax	0.5	mu6	Sat2	ENSRNOG00000011714	7.4E-01	1.0	205	1.6E-03	0.5	209

(p.adj) denotes adjusted p-value for hypoxia differential expression, (i) denotes probe intensity, Fold denotes fold change, where columns with *italics* title refer to *Spalax* and columns with plain titles refer to rat.

### Validation of microarray expression results

**Figure 9:**
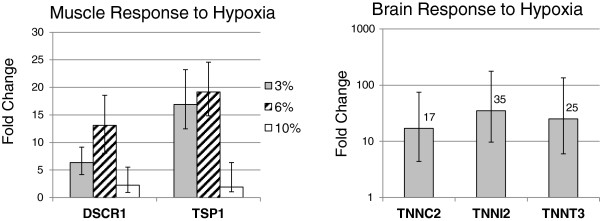
**Hypoxia induced differential expression results for antiangiogenic and contractile fiber genes, based on RT-Q-PCR.** Left: fold change (hypoxia/normoxia) for *Dscr1* and *Tsp1*antiangiogenic genes, under 3%/6%/10% hypoxia in muscle 6%. Right: fold change for *Tnnc2*, *Tnni2*, and *Tnnt3*contractile genes, under 10% hypoxia in the brain.

Real time quantitative PCR was conducted on two groups of genes of interest in order to validate the microarray results. Two genes with known functions that repress angiogenesis, *Regulator of Calcineurin* (*Rcan1*) also known as *Down Syndrome Critical Region 1* (*Dscr1*), and *Thrombospondin* (*Tsp1*), both highly upregulated in *Spalax* muscle at 6% O_2_ with no response in rat, were tested using the same samples as used for the microarray experiments together with addition biological replicates. *Rcan1* expression was induced by 6-fold, 13-fold and 2-fold under conditions of 3%, 6%, and 10% O_2_, respectively. *Tsp1* was induced by 17-fold, 19-fold, and 2-fold under the same conditions (Figure 9). Samples from a second species of *Spalax* (*S. judaei*) exposed to identical conditions were also tested for these genes by RLT-q-PCR with no significant changes between normoxic and hypoxic conditions (data not shown).

The second group of genes tested by RLT-q-PCR was chosen from the contractile and muscle fiber ontology groups. Three genes from the troponin gene family, which among other related genes showed extremely high induction uniquely in *Spalax* brain under 10% O_2_, were tested in order to confirm results. Results for *Troponin C2* (*Tnnc2*), *Troponin I2* (*Tnni2*) and *Troponin T3* (*Tnnc3*) show increased expression of 17, 35, and 25-fold, respectively, under 10% O_2_ (Figure 9). Again, *S. judaei* was tested for these genes under similar conditions, however no significant changes were observed when comparing normoxic to hypoxic conditions. We also tested the Troponin genes in *Spalax* muscle tissue. While basal normoxic levels of *Tnnc2*, *Tnni2* and *Tnnc3* were 548, 2005, and 1200-fold higher in muscle than in brain, no response to hypoxia was observed in muscle tissue. *S. galili* is known to have higher hypoxia tolerance compared with *S. judaei*, due to differences of climatic regimes in their ranges of distribution. Environmental hypoxia climaxes during winter rains and flooding, when gas solubility and permeability are restricted, and is highest in *Spalax* species living under humid climates (e.g., *S. galili*), compared with those living in more arid conditions (e.g., *S. judaei*). *Spalax* expression studies have previously demonstrated significant differences between the two species in response to hypoxia [9].

## Discussion

Here we show that different functional groups of genes are significantly overrepresented during hypoxia stress in *Spalax*. We suggest that these nonrandom patterns reflect the activation of specific physiological and molecular processes which contribute to hypoxia tolerance. We discuss how such processes, as well as individual genes, may contribute to hypoxia tolerance in *Spalax*.

### Crosstalk between angiogenesis, immune response, and apoptotic control, in *Spalax* hypoxia

**Figure 10:**
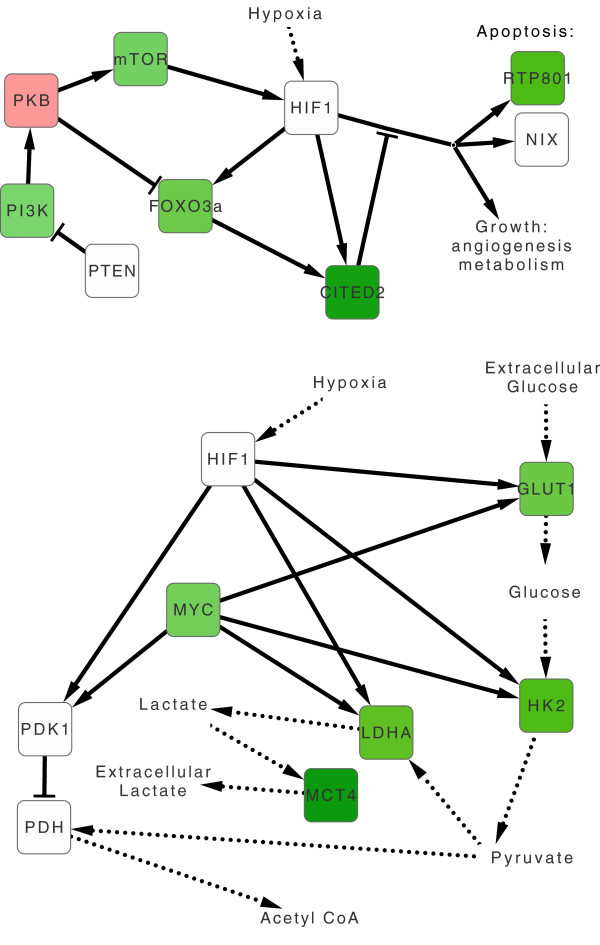
**Regulation of the FOXO3a survival, and glycolysis pathways under hypoxia in *Spalax*.** Green/red background denotes up/down regulation of *Spalax* genes under hypoxia. Top: mu6 results superimposed on the FOXO3a cell survival pathway scheme as originally published in [31]. Bottom: br6 results superimposed on the lactate synthesis and clearance pathways scheme as originally published in [32].

Processes directly involved in the formation of vascular morphology, such as angiogenesis, vasculature development, and *Vegf* signaling, were significantly enriched among hypoxia activated genes in most conditions (i.e., br3, br6, br10, mu3, mu6). In addition, we find that hypoxia activated *Spalax* genes are significantly enriched with a battery of ontologies that are known to be associated with angiogenesis including extracellular matrix (ECM), cell-cell/cell-ECM adhesion, focal adhesion, pattern binding, immune response, inflammatory response, wound healing, receptor tyrosine kinase activity, among others (see Figures 3, 4, 5, 6, 7 and 8). The ECM is known to encode chemotactic, haptotactic, and mechanotactic cues, that are crucial for the control of endothelial cell (EC) migration, and their interaction with supporting cells [29,30]. During angiogenesis the ECM also modulates EC cell survival by integration of signals induced by the binding of EC integrins to the ECM, with those induced by growth factors [30]. Multiple factors affect cell survival, including hypoxia and inflammation, which are known to modulate the balance between EC apoptosis and survival. Our results indicate that in addition to the expected apoptotic response, strong regulation of anti-apoptotic genes is observed in *Spalax* during hypoxia.For example, we find that the *Foxo3a* gene, which critically inhibits *HIF1a* induced apoptosis in mouse [31] is upregulated in mu3/6/10. Moreover, most of the well studied key genes in the *Foxo3a* cell survival pathway are differentially expressed in *Spalax* muscle, namely: *Pi3k, Pkb (Akt1), mTOR, Foxo3a, Cited2, Nix (Bnip3L),* and *RTP801* (Figure 10). This may reflect a fine balance between inducers and suppressors of apopototic pathways. Accordingly, it appears that hypoxia induced apoptosis is tightly controlled, and possibly restricted in *Spalax*. Similarly, previous studies suggested that critical p53 hypoxia induced apoptotic pathways are blocked in *Spalax*[15,16]. Also, the accepted functions assigned to genes among the common databases may not reflect the true functions of homologues in all organisms, for example, the p53 pathway as mentioned above. It was further suggested that both cancer adaptivity and hypoxia tolerance evolution require a common shift toward antiapoptotic functions. This hypothesis can be supported by different experimental results, suggesting that non-pathological internal hypoxia may be frequent during *Spalax* life. In contrast, in most adult mammals, the occurrence of internal hypoxia more frequently reflects unwanted cellular abnormalities.

### Strong elevation of key antiangiogenic genes in *Spalax* under hypoxia

The process of angiogenesis or the sprouting of new blood vessels from existing ones is controlled by a balance of pro and antiangiogenic factors [33]. Among these factors *Vegf* is probably the most studied, and elevated levels of this gene product were shown to play a critical role in the inherently dense vasculature of *Spalax* muscle. Unlike the rat and other mammals, *Spalax Vegf* remains at constant levels in muscle under hypoxic conditions and even downregulated under certain hypoxic stresses [6,17]. Our microarray data shows *Spalax Vegf* downregulation in muscle and upregulation in brain under hypoxic conditions. However, in response to hypoxia, a number of genes with known antiangiogenic function are highly expressed in *Spalax* muscle. *Thrombospondin 1* (*Tsp1*), upregulated by a factor of 19-fold in *Spalax* hypoxic muscle, was one of the first genes to be recognized as a suppressor of angiogenesis [34,35] and works through a number of mechanisms including suppression of the bioavailability of *Vegf* and inhibition of endothelial cell migration [36]. *Down Syndrome Critical Region 1* (*Dscr1* also known as *Rcan1*), a negative regulator of calcineurin and suppressor of angiogenesis lies in the region of human chromosome 21, which in the trisomic state is implicated as the major cause of Down’s Syndrome. Individuals with Down’s Syndrome have a much lower incidence of solid tumors than the general population and this is attributed to the inherently higher levels of *Dscr1* and suppresion of angiogenesis, thus the inability of tumors to develop under hypoxic conditions. In a mouse model, it was demonstrated that a single extra copy of *Dscr1* is sufficient to suppress tumor growth due to reduced tumor angiogenesis [37]. In our experiment we observed a 13-fold induction of *Dscr1* in hypoxic *Spalax* muscle under 6% hypoxia. Induction of these genes may confer the tight control of *Vegf* and angiogenic stability under hypoxic conditions in *Spalax*. Both of these genes have been proposed as potential targets for cancer therapy [38]. Recently we have proposed Spalax as a model organism for both hypoxia tolerance and cancer resistance, as we have never seen the spontaneous development of solid tumors in these animals, including many that have been in captivity for over 20 years. These genes may also play a critical role in cancer suppression in *Spalax*.

### Protective processes in *Spalax* brain under chronic hypoxia

Numerous findings indicate that the properties of the blood brain barrier (BBB) and its constituents are modulated during hypoxia and angiogenesis [30,39,40]. High altitude cerebral edema, a severe form of altitude sickness characterized by brain tissue swelling, is caused by hypoxia induced disturbance in the BBB, e.g., increased BBB permeability. Normally, the BBB maintains an ionic/molecular/volume homeostasis of brain interstitial fluids and, therefore, stabilizes synaptic/axonal signaling and blocks excessive crosstalk between different CNS compartments [40]. Among *Spalax* br10 genes, processes known to disturb BBB functions, such as inflammation, angiogenesis, and *Vegfr* signaling, are significantly overrepresented (Figure 8). Therefore, it is probable that *Spalax* developed opposing mechanisms that balance between BBB selectivity and hypoxia induced BBB permeability, which together promote brain homeostasis under hypoxia. We speculate that the observed enrichment of contractile fiber genes in br10 (Figure 8) may reflect hypoxia induced changes in the neurovascular unit cells in the BBB. Specifically, this pattern may reflect increased numbers and activities of pericytes and smooth muscle cells, which are known to express contractile fiber genes. Accordingly, hypoxia induced pericyte contraction was found to be critical in pathological processes in mouse brain [41]. The ‘contractile fiber’ group includes two upregulated genes that encode the known pericyte markers *Abcc9*, and *Kcnj8*[42]. In addition, it was found that pericytes express tropomyosins [43], of which, *Tpm2* and *Tpm4* were detected in the group of upregulated genes, and were mapped to the term ‘muscle protein’. This furthur supports the evidence of angiogenic processes with corresponding pericyte activity. Collectively, it appears that chronic mild hypoxia in *Spalax* brain induces very strong angiogenic responses in *Spalax*, and it remains to be seen how blood brain barrier homeostasis is maintained under these conditions.

### Alternate groups of TFs are expressed during hypoxia, in *Spalax*

It appears that hypoxia coordinates a transcriptional switch, in which distinct groups of TFs transcripts are suppressed, while others are increased, in *Spalax*. Accordingly, genes involved in the regulation of transcription were significantly overrepresented among both downregulated and upregulated genes, especially in brain and muscle tissues under acute hypoxia. In addition, under these conditions, bZIP TFs were found to be overrepresented, whereas C2H2 Zinc-finger TFs were found to be underrepresented. The family of bZIP TFs is characterized by a highly conserved basic region (BR), and a leucine zipper (LZ) domain that allows homo- and hetero-dimerization of bZIP monomers [44,45]. The DNA binding affinity of bZIP dimers (which constitutes the active TFs), is known to be regulated either by redox, or by phosphorylation, possibly depending on specific cystein/serine residues at position 19 of the highly conserved BR [44]. The activation of bZIPs via redox mechanisms was suggested to depend on oxidative stress, one of the major characteristics of hypoxia. Therefore, it is possible that this group of *Spalax* bZIPs is activated by oxidative stress during severe 3% hypoxia. As noted above, the upregulation of bZIP transcripts is accompanied by a decrease of C2H2-like zinc finger transcripts. In addition, a small group of C2H2-like zinc finger genes, belonging to the KRAB zinc finger family, is overrepresented among downregulated genes in br3 and mu3 (Figure 5). While we did not specifically observe activation or deactivation of these trancription factors, transcript levels of the different groups were increased or reduced under hypoxic conditions. KRAB TFs were suggested to be involved in epigenetic suppression of transcription, as part of KAP1 mediated targeting of heterochromatin protein 1 (HP1) to DNA [46]. It was suggested that this mechanism allows KRAB zinc finger TFs to ‘auto-regulate’ other zinc finger TFs [47]. It is therefore possible that the observed reduction of KRAB TF transcript levels in hypoxia reflects the involvement of processes related to epigenetic control, which may lead to long term effects (e.g., after the return to normoxia). Due to these and other long-term effects, epigenetic modifications may potentially facilitate hypoxia preconditioning and postconditioning, which has therapeutic importance in limiting ischemia/reperfusion damage [48,49].

### Overlap between hypoxia and cancer induced genes in *Spalax* muscle

The present results corroborate with the known interrelations between hypoxia and cancer conditions [18]. Hypoxia responsive genes in *Spalax* muscle are significantly enriched with cancer related genes. In mu10, the enriched term ‘pathways in cancer’ is mapped to several known genes of special importance, such as *Vegf*, *Vegfr2*, *Glut1, Egf, Fdgr, Pi3k, Fak, Foxo1, p21*, and *Myc*. Similarly, in mu3, and mu6, a group of proto-oncogenes is significantly enriched. In addition, key genes participating in glucose uptake and the lactate clearance pathway during cancer are found to be upregulated in both br3 and br6 (Figure 10) [50,51]. It is possible that lactate clearance has an anti-apoptotic role, as this mechanism reduces acidosis, a condition which may trigger apoptosis. Lactate transported out of hypoxic cells may be recycled by neighboring normoxic cells as part of angiogenesis, in both normal and cancer conditions [32]. It also appears that a group of mitochondrial genes, which includes different oxidative phosphorylation genes, is enriched among hypoxia suppressed *Spalax* genes.

### Developmental genes during hypoxia, in *Spalax*

In the present study, genes associated with different developmental processes were significantly overrepresented under hypoxia. Corresponding ontology terms include reproductive development, sexual reproduction, embryonic development, skeletal system development, face morphogenesis, wound healing, as well as angiogenesis related processes. In general, normal developmental processes are known to be tightly controlled by spatiotemporal hypoxia gradients [52]. In embryonic development, hypoxia gradients serve as essential signals that trigger a *Hif* dependent shift between different developmental stages, thereby modulating differentiation, proliferation, apoptosis, and vascularization. For example, Labyrinthine layer development, which is a process known to be specifically coordinated by hypoxia in the placenta is overrepresented among br3∩mu3 activated genes [52]. In addition, Egf like growth factors, which are overrepresented among mu3 and br10 responsive genes, may also be involved in developmental processes [53]. These growth factors were suggested to serve as potent insoluble ECM-bound *Egfr*-receptor ligands [54] and promote mitogenic activity, though their functions are diverse. Altogether, it is possible that the observed overrepresentation of development related genes reflects the control of cell proliferation vs. differentiation, during hypoxia in *Spalax*.

### Glycoproteins and disulfide-bond forming proteins during hypoxia in *Spalax*

Genes encoding membrane/transmembrane/secreted proteins, glycoproteins, disulfide-bond forming proteins, and signal peptide containing proteins, are found to be highly overrepresented under most hypoxic conditions and appear in very large numbers (see Figures 3, 4, 5, 6, 7 and 8). Glycosylated segments and disulfide-bonds are usually found in transmembrane domains, or in proteins secreted to extracellular environments [55]. The observed large overlap, between the ontologies disulfide-bond, and signal peptide, mainly reflects the role signal peptide domains play in the post-translational transport of disulfide-bond containing proteins to the rough endoplasmic reticulum, as part of their normal processing. Accordingly, very large groups of hypoxia induced genes are involved in mediating cellular interaction with the extracellular environment, which may point toward their involvement in angiogenesis, immune response, and signal transduction. As pointed out previously [21], the overrepresentation of disulfide bond forming proteins may partly reflect oxygen dependent mechanisms, as these bonds are formed by the oxidation of the thiol groups in cysteins, and are dissociated when the cellular oxygen pressure is diminished thereby leading to changes in protein conformation and activity [56,57]. It was suggested that proper folding of disulfide bond containing proteins (e.g., *Vegf*) is compromised under hypoxia, and that specific *Hif* dependent pathways increase correct protein folding and secretion [58].

## Conclusions

(1) The present study identifies multiple hypoxia induced gene and pathway responses in *Spalax*. Expression patterns of these genes reflect mechanisms of hypoxia tolerance that enhance survival in a high stress environment, with both special evolutionary and biomedical importance. Previous studies have demonstrated multiple differences between *Spalax* and rat expression patterns under hypoxia, thus, it is expected that many of the patterns observed here may be unique to *Spalax*.

(2) Expression patterns of apoptosis and angiogenesis related genes confirms previous research suggesting suppression of apoptosis for enhanced survival and the tight regulation of angiogenic factors similar to studies of cancer cells.

(3) Histological detection of proteins coded by *Spalax* hypoxia induced genes can help understand the physiological context under which hypoxia related processes act. Such tests will be specifically useful for studying transcripts mapped to enriched ontologies.

(4) The enrichment of C2H2 zinc-finger TFs, KRAB TFs, mitochondrial and ribosomal genes, among hypoxia suppressed genes, may reflect critical responses to hypoxia. As genes belonging to these groups tend to have different paralogs, better understanding of the results will require mapping contigs and microarray probes to the *Spalax* genome when it becomes available.

(5) The identification of specific genes that are highly regulated and may be critical to *Spalax* survival under conditions of stress serve as an example of the critical need to study the evolution of organisms in a wide range of diverse habitats as part of the search for candidate targets for treatment of human pathologies.

## Competing interests

The authors declare that they have no competing interests.

## Authors’ contributions

AM carried out bioinformatics analysis and data mining of the microarray data. MB designed and carried out microarray experiments. MB and AA conceived and designed the study. AK supervised informatics analysis. TH and MW provided sequences for microarray design and experiments. AM, MB, AA and AK wrote and reveiwed the manuscript. All authors read and approved the final manuscript.

## Author’ information

Joint senior authors: Aaron Avivi and Mark Band.

## Supplementary Material

Additional file 1 **Table S1.** RLT-q-PCR primers. Click here for file

Additional file 2 **Table S2.** Limma differential expression statisitcs. *Spalax* differential expression statistics for mu3/6/10 and br3/6/10, mapped to mouse genes (Ensembl gene IDs), and *Spalax* contig. Expression fold change, FDR p-value and ontology terms are presented. Click here for file

Additional file 3 **Table S3.** GO terms enriched among *Spalax* genes differentially expressed in >=1, >=2, and >=3 experiments. The table includes DAVID enrichment profiles for *Spalax* probes which map to unique reference genes (unabiguous genes) as well as all probes. Terms passing an enrichment theshold of FDR 0.05 are highlighted in bold. Click here for file

Additional file 4 **Table S4.** GO terms enriched among *Spalax* genes differentially expressed in both mu3 and br3. The table includes DAVID enrichment profiles both for all data and for filtered data (after excluding probes mapped to *Spalax* contigs with ambiguous annotations). Terms eriched with FDR 0.05 are highlighted in bold. Click here for file

Additional file 5 **Table S5.** GO terms enriched among *Spalax* genes differentially expressed in mu3, mu6, mu10, br3, br6 and br10. The table includes DAVID enrichment profiles for all data. Click here for file

Additional file 6 **Table S6.** GO terms enriched among *Spalax* genes differentially expressed in mu3, mu6, mu10, br3, br6 and br10. The table includes DAVID enrichment profiles for filtered data (after excluding probes mapped to *Spalax* contigs with ambiguous annotations). Click here for file

Additional file 7 **Table S7.** GO terms enriched among rat genes differentially expressed in mu6, and br6. The table includes DAVID enrichment profiles for all rat data. Click here for file
