# Supplementary material for: Transcriptome analysis of the spalax hypoxia survival response includes suppression of apoptosis and tight control of angiogenesis
Source: BMC Genomics. 2012 Nov 13;13:615. doi: 10.1186/1471-2164-13-615 (PMC3533650; doi:10.1186/1471-2164-13-615)
Supplement: Additional file 1 — Table S1. RLT-q-PCR primers. [file 1471-2164-13-615-S1.pdf]

Table S1: RLT-q-PCR primers.

Spalax TNNT3<br>5'- GGA GGT CGC TGG AAG TAA AGG -3' -F<br>5'- AAG TCA GGC AGG CAG GTC TCT -3' -R
Spalax TNNI2<br>5'- CAT CCC GGG CTC CAT GT -3' -F<br>5'- CGA TCT TCG CAT GCA GTT GT -3' -R
Spalax TNNC2<br>5'- GCG GCC ATC ATT GTT CTT ATC -3' -F<br>5'- GCA CGT GAC AGA CGA GGA GAT -3' -R
Spalax TSP1<br>5'- CCA TTT CAC ATG GAA TTG GTT GT -3' -F<br>5'- AGA GGG CTG TGG CTT CCT TT -3' -R
Spalax DSCR1<br>5' - TCC AGT TCT ACA AAC CGC TAG TG - 3' -F<br>5' - CGG CCT GTG GTT GTT TCC – 3' -R
18S<br>5'- GAT CCA TTG GAG GGC AAG TCT -3' -F<br>5'- AAC TGC AGC AAC TTT AAT ATA CGC TAT T -3' -R
